# Neutrophil Activation/Maturation Markers in Chronic Heart Failure with Reduced Ejection Fraction

**DOI:** 10.3390/diagnostics12020444

**Published:** 2022-02-09

**Authors:** Suriya Prausmüller, Georg Spinka, Stefanie Stasek, Henrike Arfsten, Philipp Emanuel Bartko, Georg Goliasch, Martin Hülsmann, Noemi Pavo

**Affiliations:** Department of Internal Medicine II, Division of Cardiology, Medical University of Vienna, 1090 Vienna, Austria; suriya.prausmueller@meduniwien.ac.at (S.P.); georg.spinka@meduniwien.ac.at (G.S.); stefanie.stasek@meduniwien.ac.at (S.S.); henrike.arfsten@meduniwien.ac.at (H.A.); philippemanuel.bartko@meduniwien.ac.at (P.E.B.); georg.goliasch@meduniwien.ac.at (G.G.); noemi.pavo@meduniwien.ac.at (N.P.)

**Keywords:** heart failure, neutrophil activation, CD10, CD11b, CD66b, CD64

## Abstract

Background: Neutrophils are critically involved in the immune response. Inflammatory stimuli alter the expression status of their surface molecule toolset, while inflammation-stimulated granulopoiesis might also influence their maturation status. Data on neutrophil status in heart failure with reduced ejection fraction (HFrEF) are scarce. The present study aims to evaluate the role of neutrophil CD11b, CD66b and CD64 expression in HFrEF. Methods: A total of 135 HFrEF patients and 43 controls were recruited. Mean fluorescence intensity of the activation/maturation markers CD11b, CD66b and CD64 was measured on neutrophils by flow cytometry. CD10 (neprilysin) expression was simultaneously determined. Results: Neutrophil CD64 expression was higher in HFrEF compared with controls, while CD11b/CD66b levels were similar. Neutrophil CD11b and CD66b showed a significant direct correlation to neutrophil CD10 expression (rs = 0.573, *p* < 0.001 and rs = 0.184, *p* = 0.033). Neutrophil CD11b and CD66b correlated inversely with heart failure severity reflected by NT-proBNP and NYHA class (NT-proBNP: rs = −0.243, *p* = 0.005 and rs = −0.250, *p* = 0.004; NYHA class: *p* = 0.032 and *p* = 0.055), whereas no association for CD64 could be found. Outcome analysis did not reveal a significant association between the expression of CD11b, CD66b and CD64 and all-cause mortality (*p* = ns). Conclusions: The results underline the potential role of neutrophils in HFrEF disease pathophysiology and risk stratification and should stimulate further research, characterizing subpopulations of neutrophils and searching for key molecules involved in the downward spiral of inflammation and heart failure.

## 1. Introduction

Inflammatory processes critically regulate the development, progression and outcomes of cardiovascular disease. Neutrophils are the most abundant type of white blood cells in human circulation. In contrast to monocytes and macrophages [1], they traditionally have been regarded as bystanders of cardiovascular disease, yet studies in recent years have demonstrated important functional roles of neutrophils in cardiovascular inflammation and repair [2].

Neutrophils are regarded as terminally differentiated short-lived phagocytes with a rather uncontrolled mode of action and a high turnover rate. Neutrophil production predominantly occurs in the bone marrow, where chemokines, growth factors and adhesion molecules control the release of neutrophils into the bloodstream. The half-life of neutrophils is estimated at about 12 h [3]. However, neutrophils may reside in tissues much longer, while inflammation and hypoxia even extend neutrophil lifespan up to 7 days [4,5,6]. In case of inflammation neutrophils are rapidly recruited from the bloodstream to tissue in a multistep recruitment cascade, where they might contribute to tissue damage [7]. Neutrophils, however, are equally involved in tissue healing, e.g., repairing injured intima after coronary artery injury or cardiac repair and alleviating the development of heart failure (HF) after myocardial infarction [8,9,10]. Neutrophils carry a huge toolset for instant communication with their vicinity, yet molecular patterns differ between tissues, offering the possibility for tissue-specific response and regulation [7]. Whether a steady-state neutrophil infiltration in the heart is present in states of low-grade inflammation as HF is currently unclear.

Although neutrophils have been regarded as a homogenous cell population, diverse neutrophil subpopulations with phenotypic and functional differences were reported [2]. Identification and characterization of these subpopulations are important when considering their impact on cardiovascular diseases. Once activated, neutrophils have the capacity for phagocytosis, chemotaxis, oxidative burst and can upregulate receptors and produce chemokines [11]. CD11b, CD66b and CD64 are neutrophil activation markers, with low expression in resting neutrophils but excess expression upon proinflammatory stimuli. Most recently, neutrophil activation patterns have also been studied in acute lung injury and acute respiratory distress syndrome related to coronavirus disease 2019 (COVID-19) [12].

Currently, there are no data upon the neutrophil status characterized by CD11b, CD66b and CD64 in HF patients. This study aims to provide data on the activation/maturation status and the role of circulating neutrophils in heart failure with reduced ejection fraction (HFrEF).

## 2. Materials and Methods

### 2.1. Study Population and Study Endpoint

HFrEF patients on optimal medical therapy, including angiotensin-converting enzyme inhibitor, angiotensin-receptor blocker, angiotensin receptor and neprilysin (NEP) inhibitor were enrolled prospectively at the Vienna General Hospital, a university-affiliated tertiary care center between January 2019 and March 2020. Medical history, including cardiovascular risk factors, current medication and follow-up data, were recorded. In accordance with the HF guidelines of the European Society of Cardiology, HFrEF was defined as a history of signs and symptoms of HF and formerly documented left ventricular ejection fraction below 40% [13]. All-cause mortality was chosen as the primary outcome parameter. In addition, forty-three apparently healthy control subjects, without previously recorded comorbidities or medication, were included. The study was approved by the local Ethics Committee and performed according to the current revision of the Helsinki Declaration.

### 2.2. Sampling and Routine Laboratory Analysis

Venous blood samples were drawn from all participants on the day of study inclusion. Routinely available laboratory parameters were analyzed according to the local standards of the Department of Laboratory Medicine of the Medical University of Vienna.

### 2.3. Determination of Neutrophil Cell Surface Markers by Flow Cytometry

Mean fluorescence intensity (MFI) of the neutrophil surface markers CD11b, CD66b and CD64 was measured on peripheral neutrophils by flow cytometry. Additionally, MFI of CD10, i.e., neutrophil NEP, was determined. The results of neutrophil NEP expression have been published separately [14]. Freshly collected whole blood samples were stored at 4 °C and were processed within 4 h after the specimens were collected. A measure of 100 μL of EDTA-anticoagulated blood was stained with the following antibodies: CD16 [#335035], CD10 [#332777], CD45 [#560178], CD11b [#555388], CD64 [#561191] and CD66b [#562254] (BD Biosciences, San Jose, CA, USA), as described earlier [14]. All samples were incubated in the dark for 30 min, followed by red blood lysis using FACS lysing solution (BD Biosciences). Then, cells were washed twice with phosphate-buffered saline (PBS) solution and finally the pellet was resuspended in 500 µL of PBS. For each sample, at least 30,000 events were recorded. Neutrophils were gated as FSC^high^SSC^high^CD16^+^CD45^+^ populations. Fluorescence, minus one control tube, were used to determine positive fluorescence.

### 2.4. Statistical Analysis

Categorical data were presented as counts and percentages, and continuous data as median and interquartile ranges (IQR). Categorical variables were compared by the Fisher’s exact test, and continuous variables were compared by the Kruskal–Wallis and Mann–Whitney U tests. The correlation between neutrophil surface markers and laboratory parameters was assessed by calculating Spearman’s rho correlation coefficient and displayed using scatter plots. Kaplan–Meier curves using log-rank test were generated to graphically illustrate the association of the neutrophil activation/maturation markers with the endpoint. Cox proportional hazard regression analysis was applied to evaluate the association between the respective neutrophil activation/maturation parameters and the predefined endpoint. Results are presented as hazard ratios (HR) per IQR and 95% confidence intervals (CI). To account for potential confounding effects, we formed a clinical confounder cluster encompassing age, kidney function and sex. All tests were two-sided and a *p*-value < 0.05 was considered to indicate statistical significance. Statistical analysis was performed using RStudio (R Foundation for Statistical Computing, Vienna, Austria) version 1.3.1073 and SPSS software (IBM SPSS, Chicago, IL, USA) version 24.

## 3. Results

### 3.1. Baseline Characteristics

A total of 135 HFrEF patients were included in the study. Table 1 displays the detailed baseline characteristics of the study population. Median age was 64 years (56–72), 101 (75%) of the patients were male. Most patients were in New York Heart Association (NYHA) class II (44%) and III (48%) and median NT-proBNP levels were 2107 pg/mL (IQR 745–4407). HF therapy was well established with 124 (92%), 128 (95%) and 107 (79%) study participants receiving renin–angiotensin system blockade, beta-blockers and mineralocorticoid receptor antagonist therapy, respectively. Neutrophil CD10/CD11b/CD66b/CD64 expression was not different in patients receiving angiotensin receptor neprilysin inhibitors compared with patients receiving angiotensin-converting enzyme inhibitors/angiotensin II receptor blockers (*p* = 0.066 for CD10, *p* = 0.801 for CD11b, *p* = 0.053 for CD66b, *p* = 0.539 for CD64).

### 3.2. Expression of Neutrophil Activation/Maturation Markers in Heart Failure Compared with Controls

Supplementary Figure S1 displays the expression patterns of the neutrophil activation/maturation markers CD11b, CD66b and CD64 in HFrEF patients and controls. Neutrophil CD64, expressed as MFI, was significantly higher in HFrEF patients as compared with controls (5131 [4461–6039] vs. 4514 [4095–5031], *p* < 0.001), whereas no significant difference could be found for CD11b and CD66b (30212 [23099–39710] vs. 29612 [21248–36581], *p* = 0.310 and 5103 [4162–6435] vs. 4613 [3677–5626], *p* = 0.179, respectively).

### 3.3. Correlation between Neutrophil Activation/Maturation Markers and Neutrophil Neprilysin

**Figure 1:**
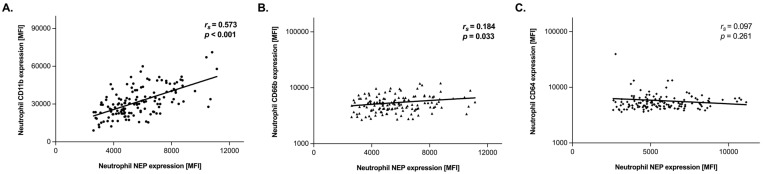
Relationship between neutrophil NEP expression and neutrophil activation/maturation markers CD11b (**A**), C66b (**B**) and CD64 (**C**). Scatter plot with linear regression analysis was performed and fit curves are shown. Spearman’s correlation coefficient and level of significance are indicated in the respective plots.

Neutrophil NEP expression (CD10) correlated directly and highly significantly with CD11b expression (rs = 0.573, *p* < 0.001) and moderately with CD66b (rs = 0.184, *p* = 0.033). There was no correlation between neutrophil NEP and CD64 (rs = 0.097, *p* = 0.261). The respective scatterplots are illustrated in Figure 1.

### 3.4. Association of Neutrophil Activation/Maturation Makers with Heart Failure Severity

**Figure 2:**
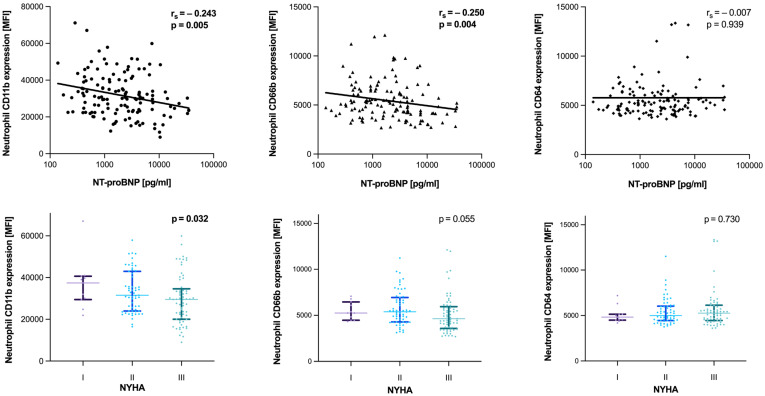
Relationship of neutrophil activation/maturation markers and heart failure severity. Scatter plots with linear regression analysis and the Spearman’s rho correlation coefficient for mean fluorescence intensities (MFI) of neutrophil CD11b, CD66b and CD64 expression with N-terminal pro B-type natriuretic peptide (NT-proBNP), as well as group comparisons between New York Heart Association (NYHA) class, are shown. Comparison between groups has been assessed by using the Kruskal–Wallis test, level of significance is indicated in the respective plots.

Figure 2 shows the association of the neutrophil makers CD11b, CD66b and CD64 with HF severity, reflected by NT-proBNP and NYHA class. Both, neutrophil CD11b and CD66b expression correlated inversely with NT-proBNP concentration (rs = −0.243, *p* = 0.005; rs = −0.250, *p* = 0.004; respectively). Additionally, there was an inverse association between neutrophil CD11b expression and NYHA class (*p* = 0.032), and a trend for CD66b (*p* = 0.055). No obvious relationship could be demonstrated for neutrophil CD64 expression and HF severity HF.

### 3.5. Association of Neutrophil Activation/Maturation Markers with Clinical and Laboratory Parameters

The neutrophil surface markers were comparable for both sexes (CD11b: *p* = 0.879; CD66b: *p* = 0.659; CD64: *p* = 0.303). CD11b showed no correlation with age or body mass index (BMI), CD66b showed an inverse correlation with age (rs = −0.181, *p* = 0.036) and a direct correlation with BMI (rs = 0.243, *p* = 0.005) and CD64 showed a weak correlation with BMI (rs = 0.174, *p* = 0.044) but not age. There was no meaningful correlation between CD11b, CD66b or CD64 expression and liver functional parameters (aspartate aminotransferase: CD11b: rs = −0.025, *p* = 0.775; CD66b: rs = 0.060, *p* = 0.492; CD64: rs = 0.054, *p* = 0.540; alanine aminotransferase: CD11b: rs = −0.021 *p* = 0.815; CD66b: rs = 0.109, *p* = 0.212; CD64: rs = 0.114, *p* = 0.192), inflammatory parameters (C-reactive protein: CD11b: rs = 0.011, *p* = 0.901; CD66b: rs = 0.031, *p* = 0.727; CD64: rs = 0.119, *p* = 0.178) or kidney function (creatinine: CD11b: rs = −0.111, *p* = 0.200; CD66b: rs = −0.215, *p* = 0.012; CD64: rs = 0.113, *p* = 0.191).

### 3.6. Neutrophil Activation/Maturation Makers and Outcome

**Figure 3:**
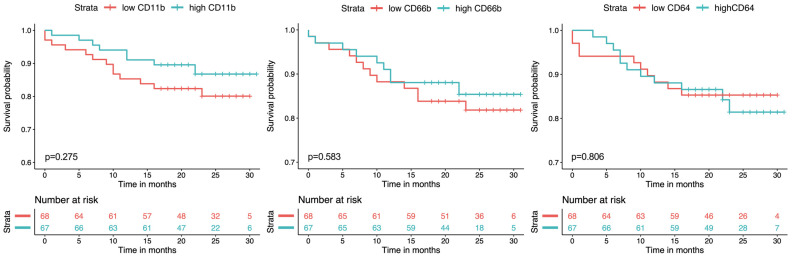
Kaplan–Meier analysis for the outcome all-cause mortality with low and high expression of neutrophil activation/maturation markers with the median mean fluorescence intensity (MFI) as the cut-off value. Comparison was calculated by the log-rank test.

During the median follow-up period of 22 months (IQR 18–27), the endpoint all-cause death was reached in 21 (16%) HFrEF patients. Univariate Cox regression analysis revealed no significant association between neutrophil CD11b, CD66b and CD64 expression and the endpoint all-cause death (*p* = ns for all). Detailed results of the univariate and multivariate Cox regression analysis of the respective markers are displayed in Table 2. The association of neutrophil activation/maturation markers and outcome is graphically illustrated in Figure 3.

## 4. Discussion

The data reported in this study represent the first description of activation/maturation status of peripheral neutrophils in chronic HFrEF patients. Neutrophil CD64 expression was higher in HFrEF compared with controls. Neutrophil CD11b and CD66b correlated inversely with HF severity reflected by NT-proBNP and NYHA class, whereas no association for CD64 could be found. Additionally, neutrophil CD11b and CD66b showed a significant direct correlation with neutrophil CD10 (neprilysin) expression, while higher levels of CD10 have been associated with better outcome previously [14]. Outcome analysis did not reveal a significant association between the expression of CD11b, CD66b and CD64 and all-cause mortality in this population.

Analogous to sustained neurohumoral activation, chronic subclinical inflammation plays a critical role in the development and pathology of HF [15]. Chronic HF is known to activate the immune system and inflammatory responses characterized by elevated levels of circulating proinflammatory cytokines [15,16]. Inflammation and immune cells participate in both acute myocyte injury and HF. Inflammatory factors, such as TNF-alpha, interleukin-1beta, interleukin-6 and lectin 3, are increased in HF patients [17]. Small animal models of myocarditis and pressure overload suggest an immune activation during HF [18]. Yet, clinical trials based on anti-inflammatory or immunomodulatory therapies have yielded disappointing results, except for canakinumab or anakinra in special subpopulations [15,19]. The failure of previously attempted anti-inflammatory therapies underlines the complexity of the immune system’s role in chronic HF. Several clinical studies targeting the chemokines involved in neutrophil trafficking are ongoing.

Neutrophils are the most abundant type of white blood cells in human circulation with a vast turnover. In chronic inflammation, inflammation-driven myelopoiesis introduces functional disturbances of the hematopoietic stem and progenitor cells (HSPC) to be able to sustain neutrophil numbers. Additionally, lipid and glucose metabolism as well as ageing essentially regulate myelopoiesis and neutrophil function. Cardiovascular risk factors, such as hypercholesterinemia, hyperglycemia, age and stress, reprogram HSPC function and myelopoiesis to generate primed proinflammatory neutrophils [2]. Stress-induced neutrophilia may lead to enhanced infiltration of atherosclerotic lesions by neutrophils thereby accelerating atherosclerosis and leading to plaque instability [20]. Similarly, induction of myocardial pressure overload in a mouse model resulted in rapid influx of neutrophils into the heart; whereas, the induction of neutropenia alleviated cardiac hypertrophy and dysfunction and reduced inflammation [21].

Neutrophils are not a homogenous cell population with mature and diversified cells but diverse neutrophil subpopulations with phenotypic and functional differences [2]. Characterization of these subpopulations are important to correctly interpret their role in cardiovascular diseases.

CD11b, also known as integrin alpha-M and macrophage-1 antigen, is an integrin family member that is involved in adhesion to activated endothelial cells at sites of inflammation and other immune processes. Upon activation, integrin alpha-M/beta 2 binds to several ligands, including ICAM-1, fibrinogen and the C3 complement fragment C3bi, to mediate phagocyte adhesion, migration and ingestion of complement-opsonized particles. CD11b is upregulated by lipopolysaccharide stimulation [22]. Moreover, neutrophil CD11b expression was suggested as an early marker of early onset neonatal infection [23].

CD64, also called Fc gamma receptor 1, is a class of plasma membrane receptors on human myeloid cells. In resting neutrophils, CD64 is expressed at very low levels; upon neutrophil activation it is strongly upregulated by the proinflammatory cytokines interferon gamma and granulocyte colony-stimulating factor, which are produced during infections or exposure to endotoxins [24]. Expression of the Fc receptor CD64 on their surface has been shown to correlate with complications in sepsis, infectious diseases and solid organ transplant recipients [25,26,27].

CD66b (CEACAM8, CGM6, NCA-95) is a single chain glycosylphosphatidylinositol anchored protein and a member of the immunoglobulin superfamily, more specifically, the human carcinoembryonic antigen family [28]. CD66b is exclusively expressed on human granulocytes and is recognized as a granulocyte activation marker [29]. Neutrophil CD66b overexpression has been reported in the context of staphylococcus aureus induced neutrophil dysfunction [30].

Thus, CD11b, CD66b and CD64 are neutrophil activation markers, with low expression in resting neutrophils but excess expression upon proinflammatory stimuli. In the current report we did not observe an association between CD11b, CD66b and CD64 and outcome; however, with increasing CD11b expression, we noticed a trend towards improved survival. The limited number of events may disallow the detection of a significant association with outcome, which could be especially worthwhile to investigate for CD11b in future studies. As HFrEF is characterized by a low-grade chronic inflammation, increased levels of neutrophil activation markers might have been anticipated in HF patients. However, low-grade inflammation present in HF is different from acute infection and may result in different effects on neutrophil turnover and status. CD11b and CD66b may also be regarded as maturation markers. Low CD11b expression is one marker for immature low-density neutrophils, found in the blood of patients with inflammatory diseases or malignancies [31]. Similarly, a massive mobilization of immature neutrophils, characterized by low CD10 expression, has been described during severe systemic infections [32], which could go along with the direct association found between CD11b/CD66b and CD10 expression found within this study. The immunoregulatory properties of immature neutrophils were discussed, controversially, and remain unclear. In the current report, CD64 was expressed at higher levels in HFrEF patients as compared to controls. CD64 has gained a lot of attention in recent years as an inflammatory marker and has been discussed as an immunomodulatory target for chronic inflammatory diseases [33]. In the context of HF, CD64 may reflect an enhanced low-grade proinflammatory state. 

Recently neutrophil activation patterns have also been explored in COVID-19 [12]. While CD64 and CD66b were increased, CD11b was not altered on otherwise activated neutrophils, suggesting an immune dysfunction in COVID-19. In light of these observations, preexisting changes in neutrophil activation patterns may predispose HF patients to worse outcomes under COVID-19 infection.

## 5. Conclusions

Among the neutrophil activation/maturation markers CD11b, CD66b and CD64, only CD64 is increased in chronic HF compared with healthy controls. In contrast, CD11b and CD66b expression correlate directly with neutrophil CD10 (neprilysin) expression and inversely with HF severity, reflected by NT-proBNP and NYHA class, whereas CD64 does not. CD11b/CD66b and CD64 seem to indicate distinct changes in neutrophil status in HFrEF, CD64 assumedly mirroring an enhanced low-grade proinflammatory state, while decreased CD11b/CD66b expression indicating altered neutrophil maturation status. The results underline the potential role of neutrophils in HFrEF disease pathophysiology and risk stratification and should stimulate further research within this field, characterizing subpopulations of neutrophils and searching for key molecules involved in the downward spiral of inflammation and HF.

## Figures and Tables

**Table 1:** Baseline characteristics of the total study population (*n* = 135). Continuous variables are given as median and interquartile range, counts are given as numbers and percentages.

Baseline Characteristics	Total Study Population (*n* = 135)
Age, years (IQR)	64 (56–72)
Male sex, n (%)	101 (75)
BMI, kg/m^2^ (IQR)	28 (24–31)
Systolic blood pressure, mmHg (IQR)	120 (105–130)
Diastolic blood pressure, mmHg (IQR)	72 (70–83)
Heart rate, min^−^¹ (IQR)	69 (61–80)
NYHA functional class	
NYHA I, n (%)	11 (8)
NYHA II, n (%)	59 (44)
NYHA III, n (%)	65 (48)
NYHA IV, n (%)	0 (0)
**Comorbidities**	
Non-ischemic etiology of HF, n (%)	66 (49)
Hypertension, n (%)	87 (64)
Type II diabetes mellitus, n (%)	52 (39)
Atrial fibrillation, n (%)	53 (39)
**Laboratory parameters**	
Hemoglobin, g/dL (IQR)	13.6 (12.2–14.3)
WBC, G/l (IQR)	7.05 (5.95–8.75)
Serum creatinine, mg/dL (IQR)	1.24 (0.90–1.77)
Blood urea nitrogen, mg/dL (IQR)	32.5 (16.9–35.1)
Total cholesterol, mg/dL (IQR)	164 (128–190)
C-reactive protein, mg/dL (IQR)	0.30 (0.15–0.82)
BChE, U/I (IQR)	6.87 (5.49–8.65)
NT-proBNP, pg/mL (IQR)	2107 (745–4407)
**Neutrophil marker**	
Neutrophil CD10, MFI (IQR)	5381 (4302–6968)
Neutrophil CD11b, MFI (IQR)	30212 (23099–39710)
Neutrophil CD66b, MFI (IQR)	5103 (4162–6435)
Neutrophil CD64, MFI (IQR)	5131 (4461–6039)
**Medication**	
Beta-blocker, n (%)	128 (95)
Diuretics, n (%)	67 (50)
Mineralocorticoidantagonist, n (%)	107 (79)
I_f_ Inhibitor (%)	12 (9)
ACE-I/ARB/ARNI, n (%)	46/17/61 (34/13/45)

IQR—interquartile range; BMI—body mass index; NYHA—New York Heart Association; HF—heart failure; WBC—white blood count; BChE—butyrylcholinesterase; NT—proBNP, N-terminal pro-B-type-natriuretic peptide; MFI—mean fluorescence intensity; ACE-I—angiotensin-converting enzyme inhibitor; ARB—angiotensin II receptor blocker; ARNI—angiotensin receptor neprilysin inhibitor.

**Table 2:** Univariate and multivariate Cox regression analysis of the association between the neutrophil markers CD11b, CD66b and CD64 and all-cause mortality.

	Univariate Analysis				Multivariate Analysis		
	IQR	HR	95% CI	*p*-Value	Adj. HR *	95% CI	*p*-Value
Neutrophil CD11b	16611	0.550	0.269–1.128	0.103	0.599	0.295–1.215	0.155
Neutrophil CD66b	2273	0.864	0.499–1.495	0.601	1.042	0.594–1.827	0.887
Neutrophil CD64	1578	0.983	0.790–1.227	0.880	0.964	0.788–1.180	0.722

HR—hazard ratio; IQR—interquartile range; CI—confidence interval. * Adjusted for age, sex and creatinine.

## Data Availability

The data supporting the findings of this study can be made available from the corresponding author upon reasonable request.

## Supplementary Materials

The following are available online at https://www.mdpi.com/article/10.3390/diagnostics12020444/s1, Figure S1: Neutrophil CD11b, CD66b and CD64 expression in controls and HFrEF patients.
