# Supplementary material for: Neutrophil Activation/Maturation Markers in Chronic Heart Failure with Reduced Ejection Fraction
Source: Diagnostics (Basel). 2022 Feb 9;12(2):444. doi: 10.3390/diagnostics12020444 (PMC8871325; doi:10.3390/diagnostics12020444)
Supplement: Supplementary file 1 [file diagnostics-12-00444-s001.zip › diagnostics-1514167-supplementary.pdf]

diagnostics

MDPI

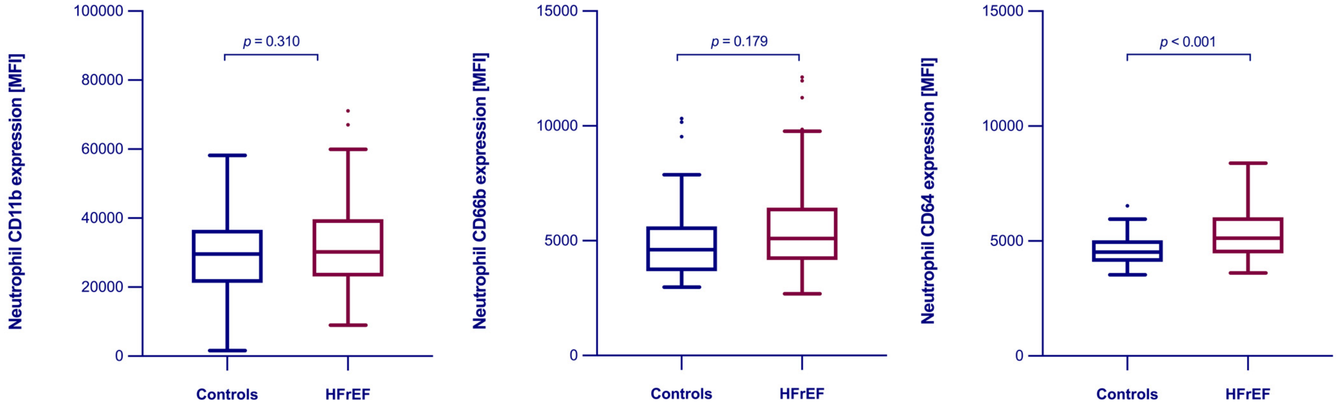

Figure S1. Neutrophil CD11b, CD66b and CD64 expression in controls and in patients with heart failure with reduced ejection fraction (HFrEF). Comparison between groups has been assessed by the Mann-Whitney-U test.
